# Two Consecutive Days of Low-Dose Methotrexate Toxicity: A Diagnostic Challenge

**DOI:** 10.7759/cureus.86023

**Published:** 2025-06-14

**Authors:** Hridya Harimohan, Quynh Huynh, Mia Yasonova, Leila Moosavi, Melanie Khamlong, Igor Garcia Pacheco

**Affiliations:** 1 Internal Medicine, Kern Medical, Bakersfield, USA; 2 Rheumatology, Kern Medical, Bakersfield, USA

**Keywords:** leucovorin, methotrexate, mucosal blisters, pancytopenia, toxicity

## Abstract

Methotrexate is an immunosuppressive medication commonly used to treat rheumatological disorders, primarily by inhibiting the folic acid cycle, with dose-dependent toxicity affecting multiple organ systems. A 54-year-old woman with a history of rheumatoid arthritis (RA), previously treated with methotrexate but later switched to leflunomide, etanercept, and prednisone, presented to the emergency department due to abnormal lab results. After running out of leflunomide and experiencing increased joint pain, she resumed methotrexate for two consecutive days without folic acid supplementation. Three days later, she developed oral ulcers, blisters, decreased oral intake, and fatigue. Lab results revealed pancytopenia, with markedly low white blood cells, hemoglobin, platelets, and absolute neutrophil count. Initially, Stevens-Johnson Syndrome (SJS) was considered due to mucosal symptoms, but lack of rash made methotrexate toxicity more likely. Rheumatology and hematology consultations led to the discontinuation of methotrexate, administration of filgrastim and leucovorin, and subsequent clinical improvement. This case highlights the diagnostic challenge in differentiating methotrexate toxicity from SJS, as both can present with mucosal lesions, though pancytopenia pointed toward toxicity. Despite methotrexate's known dose-dependent toxicity, this patient's reaction at a low dose suggests a rare idiosyncratic response, underscoring the importance of vigilance even with standard dosing and the necessity of folic acid supplementation to reduce adverse effects.

## Introduction

Methotrexate is a cornerstone disease-modifying antirheumatic drug (DMARD) used in the treatment of various autoimmune conditions, including rheumatoid arthritis (RA), psoriasis, and certain malignancies. It exerts its immunosuppressive and anti-inflammatory effects primarily by inhibiting dihydrofolate reductase, an enzyme involved in the folic acid pathway, leading to impaired DNA synthesis and cellular replication, particularly in rapidly dividing cells such as those in the bone marrow and gastrointestinal tract [1,2]. To mitigate its toxic effects, particularly on hematopoietic and mucosal tissues, concurrent folic acid supplementation is routinely recommended.

Although methotrexate is generally well-tolerated at low weekly doses used in rheumatologic diseases, it can cause serious adverse effects, particularly when dosing errors occur or folate supplementation is omitted. These toxicities may include myelosuppression, hepatotoxicity, gastrointestinal ulceration, nephrotoxicity, and neurotoxicity. Myelosuppression resulting in pancytopenia is one of the most severe and potentially life-threatening complications, and is typically dose-dependent; however, rare idiosyncratic reactions may occur even at standard or low doses [3].

Dermatologic manifestations of methotrexate toxicity, such as oral mucositis, ulcers, or blistering lesions, may resemble Stevens-Johnson Syndrome (SJS) or toxic epidermal necrolysis (TEN). Distinguishing between these entities is clinically important, as management strategies differ: methotrexate toxicity responds to folinic acid (leucovorin) rescue therapy, while SJS/TEN requires immediate withdrawal of the offending agent and supportive care [4].

This report describes a rare case of low-dose methotrexate toxicity in a patient with RA who developed severe pancytopenia and mucocutaneous lesions after reinitiating methotrexate without folic acid supplementation. The case highlights the diagnostic challenge in differentiating methotrexate toxicity from SJS and underscores the importance of maintaining a high index of suspicion even with low-dose regimens.

## Case presentation

A 54-year-old woman with a known history of seropositive rheumatoid arthritis (RA) presented to the emergency department following a referral from her primary care provider due to abnormal laboratory results (Table 1). She had been diagnosed with RA five years earlier and was initially managed with methotrexate. The methotrexate was later discontinued for unclear reasons, and she was subsequently transitioned to a regimen that included daily oral leflunomide, subcutaneous etanercept injections, and a tapering dose of prednisone (20 mg daily).

**Table 1:** Laboratory markers BUN: blood urea nitrogen; eGFR: estimated glomerular filtration rate; ALT: alanine aminotransferase; AST: aspartate aminotransferase; INR: International Normalized Ratio; PTT: partial thromboplastin time

Laboratory study	Value	Reference range
Sodium	139	135 – 145 mmol/L
Potassium	3.1	3.5 – 5.0 mmol/L
Chloride	108	98 – 107 mmol/L
Bicarbonate	24	22 – 29 mmol/L
Calcium	9.0	8.5 – 10.5 mg/dL
BUN	17	7 – 20 mg/dL
Glucose	111	70 – 99 mg/dL
Creatinine	1.15	0.6 – 1.3 mg/dL
eGFR	64	> 60
Albumin	2.8	3.4 – 5.4 g/dL
Alkaline phosphatase	74	44 – 147 IU/L
ALT	31	10 – 40 IU/L
AST	17	7 – 56 IU/L
Bilirubin direct	0.2	0.0 – 0.3 mg/dL
Bilirubin total	0.7	0.1 – 1.2 mg/dl
Prothrombin time	12	11 – 13.5 seconds
INR	0.9	0.8 – 1.2
PTT	28	25-35 seconds

Approximately one month prior to the presentation, the patient ran out of her leflunomide prescription and was unable to obtain a refill. As a result, she began experiencing worsening joint pain, which prompted her to restart methotrexate on her own. She took methotrexate at a dose of 7.5 mg daily for two consecutive days, without concurrent folic acid supplementation.

**Figure 1:**
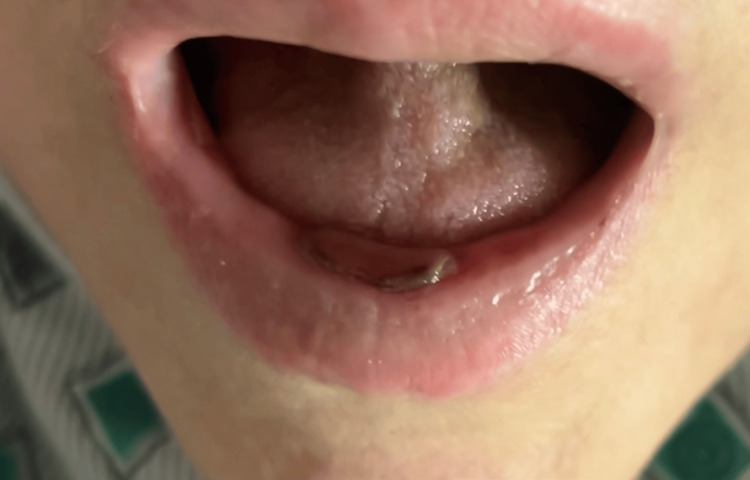
Mucosal blister in the oral cavity at the time of presentation

Three days after restarting methotrexate, the patient developed painful oral ulcers and blistering lesions localized to the oral mucosa (Figure 1). These symptoms were accompanied by progressively worsening fatigue and decreased oral intake. She denied fever, skin rash, conjunctival injection, or genital lesions. The patient did not have any other organ disorders, including renal dysfunction.

Laboratory investigations at her primary care clinic revealed pancytopenia, prompting immediate referral to the emergency department. On presentation, vital signs were stable, and physical examination was notable for oral mucositis with ulcerations but no cutaneous rash or epidermal detachment. There was no hepatosplenomegaly or lymphadenopathy.

The differential diagnosis included methotrexate toxicity and SJS. SJS was initially considered due to the presence of oral mucosal lesions; however, the absence of widespread skin involvement and systemic symptoms made this diagnosis less likely. Given the recent re-initiation of methotrexate and lack of folic acid supplementation, methotrexate-induced pancytopenia and mucositis were strongly suspected.

Methotrexate was immediately discontinued. Hematology and rheumatology consultations were obtained. The patient was treated with four doses of filgrastim (G-CSF) and initiated on leucovorin (folinic acid) rescue therapy. Supportive care, including intravenous fluids and neutropenic precautions, was provided.

The patient’s clinical condition gradually improved over the course of several days. Her oral intake increased and repeat laboratory testing demonstrated a progressive rise in white blood cell, hemoglobin, platelet, and neutrophil counts (Table 2). No new mucocutaneous lesions developed, and the existing oral lesions began to resolve. She was discharged in stable condition with hematology and rheumatology follow-up.

**Table 2:** Laboratory results

Laboratory test​	Day-1​	Day-2​	Day-3​	Day-4​	Reference range​
White Blood Count​ (x 10⁹/L​	1.8​	1.6​	2.3​	9.3​	4.0 to 11.0
Absolute Neutrophil Count (x 10⁹/L)​	-​	0.1​	0.2​	4.2​	1.5 to 8.0
Hemoglobin grams per deciliter (g/dL)​	9.6​	8.3​	7.9​	8.4​	12.1 to 15.1​
RBC (million cells per microliter (cells/mcL)​	3.01​	2.59​	2.48​	2.34​	4.2 to 5.4
Platelets​ (x 10⁹/L)​	15​	58​	66​	212​	150 to 450​

## Discussion

The two main differential diagnoses in this case were methotrexate toxicity and SJS, both of which can present with mucocutaneous findings. Methotrexate, a folate antagonist, is widely used in the management of autoimmune diseases such as rheumatoid arthritis due to its anti-inflammatory and immunosuppressive properties. However, it carries a risk of significant toxicity, particularly in the absence of folic acid supplementation [5].

SJS and its more severe form, TEN, are rare but serious mucocutaneous hypersensitivity reactions often triggered by medications, including methotrexate. These conditions are typically characterized by diffuse erythematous or purpuric macules, epidermal detachment, and mucosal involvement, most commonly affecting the oral, ocular, and genital mucosa. In contrast, methotrexate toxicity typically presents with oral mucositis, gastrointestinal disturbances, and myelosuppression, including pancytopenia, especially when folate deficiency is present [6].

In this case, the absence of skin rash or epidermal detachment made SJS less likely. Instead, the development of oral ulcers, severe neutropenia, thrombocytopenia, and anemia pointed toward methotrexate-induced myelosuppression. Notably, the temporal relationship between methotrexate reintroduction and symptom onset, along with the absence of folic acid supplementation, strengthened the diagnosis of methotrexate toxicity [7].

Folic acid plays a critical role in mitigating methotrexate toxicity by replenishing intracellular folate stores and reducing the severity of mucositis and cytopenias. Leucovorin (folinic acid), the active form of folic acid, is the recommended antidote in cases of methotrexate toxicity and can dramatically improve outcomes when administered promptly.

Importantly, methotrexate toxicity is generally dose-dependent and more commonly observed in high-dose therapy used in oncology settings. However, idiosyncratic reactions or enhanced susceptibility in certain individuals can lead to severe toxicity even at low doses, as seen in this case [8]. Factors that may contribute to increased sensitivity include renal impairment, hypoalbuminemia, drug interactions, and genetic polymorphisms affecting methotrexate metabolism [9].

This case is particularly notable because the patient received a low dose of methotrexate (7.5 mg daily for two days), a dose considered standard and well-tolerated in rheumatologic practice. Despite this, she developed profound pancytopenia and mucositis, highlighting the unpredictable nature of methotrexate toxicity in the absence of folic acid. The rapid improvement following the administration of leucovorin and filgrastim (G-CSF) supports the diagnosis and demonstrates the efficacy of current management protocols [10,11].

Clinicians must maintain a high index of suspicion for methotrexate toxicity in patients presenting with mucosal lesions and cytopenias, even if the dosage is within a typical therapeutic range. Routine patient education, monitoring of complete blood counts, and strict adherence to folic acid supplementation are essential strategies to prevent adverse outcomes [10].

## Conclusions

This case highlights the potential for severe methotrexate toxicity, including pancytopenia and mucositis, even at low doses and with short-term exposure, particularly in the absence of folic acid supplementation. The clinical presentation overlapped with features of Stevens-Johnson Syndrome, making early diagnosis challenging. However, the presence of profound cytopenias and the absence of widespread cutaneous involvement favored the diagnosis of methotrexate-induced myelosuppression. Prompt recognition and intervention with leucovorin rescue and supportive care, including filgrastim, led to rapid clinical improvement. This case underscores the need for close monitoring of blood counts, strict adherence to folate supplementation, and thorough patient education, even when methotrexate is used at standard doses for rheumatologic conditions. Clinicians must maintain a high index of suspicion for toxicity in any patient presenting with mucosal lesions and cytopenia while on or recently exposed to methotrexate.
